# Prevalence of Analgesic Prescriptions among Patients with Cancer in Japan: An Analysis of Health Insurance Claims Data

**DOI:** 10.5539/gjhs.v4n6p197

**Published:** 2012-10-12

**Authors:** Takahiro Higashi, Tetsusuke Yoshimoto, Motohiro Matoba

**Affiliations:** 1 Department of Public Health/Health Policy, University of Tokyo, Tokyo, Japan; 2 Department of Palliative and Supportive Care, Social Insurance Chukyo Hospital, Nagoya, Japan; 3 Department of Palliative Medicine, National Cancer Center Hospital of Japan, Tokyo, Japan

**Keywords:** analgesic prescription, pain management, performance measurement, cancer, opioid

## Abstract

**Objectives:**

To promote effective management of cancer pain as a nationwide health policy, it is necessary to monitor the performance of health care providers in managing pain in their patients. To plan a system that monitors the performance of pain management, the exact methods of measurement, including the range of target patients, and estimate the resources must be defined. Performance in pain management can be evaluated either in all patients with cancer or restricted to patients with cancer who are already taking analgesics. Restricting the target patient group to patients on analgesics may be more efficient but the extent of that efficiency remains uncertain.

**Methods:**

Using insurance claims from eight employer-sponsored insurance companies, we analyzed data from patients (N = 2858) who had received anti-cancer treatment (ie, surgery, chemotherapy, and radiation therapy) for the five major cancers in Japan (ie, breast, colorectal, liver, lung, and stomach cancers).

**Results:**

Overall, 22.9% of patients received some kind of analgesic prescription in the course of a month. Lung cancer patients were more likely to be prescribed analgesic prescriptions (any analgesics 34.8%; opioids 18.2%) than patients with the other four cancers. The observed percentage of patients who received analgesic prescriptions over the study period (ie, January 2005 to November 2009) decreased.

**Conclusion:**

If we limit the target patient group to patients with cancer already on analgesics, we can reduce the number of persons to be contacted by about three-fourths, compared to assessing pain in all patients with cancer. Although we do not wish to ignore the problem of undetected pain among patients with cancer, beginning our systematic evaluation with patients with cancer already on analgesics may be a realistic option.

## 1. Introduction

While pain is the most focused-on part of palliative care in cancer patients (Portenoy, 2011), management of pain is reportedly inadequate in many settings (Cleeland et al., 1994; Deandrea, Montanari, Moja, & Apolone, 2008; Okuyama et al., 2004; Uki, Mendoza, Cleeland, Nakamura, & Takeda, 1998). Few studies have examined the adequacy of pain management in cancer care in Japan (Okuyama et al., 2004; Uki, Mendoza, Cleeland, Nakamura, & Takeda, 1998). Even though cancer is the leading cause of death (Ministry of Health, 2010) in Japan, opioid consumption is relatively small compared to opioid consumption in other industrialized counties. According to a report by the International Narcotics Control Board, opioid consumption in Japan is the lowest among the G7 countries (The International Narcotics Control Board, 2010).

Concern over low opioid consumption in Japan has led policy makers to pay extra attention to pain control. The Cancer Control Act of 2007, which delegated comprehensive responsibility for cancer control to the Japanese government, specifically states that both national and local governments should “take measures to enable palliative care, such as pain control, from the early stages of cancer care processes” (Japan Law Data Archives, 2006). And The Basic Plan to Promote Cancer Control Programs established adequate pain control as a central agenda (Ministry of Health, 2007).

One way to foster adequate pain management in hospitals throughout Japan would be to establish a system to monitor their pain management programs. Measurement and feedback of hospital performance of pain management, preferably in comparison to other medical facilities, would motivate hospitals to improve their pain management (Hibbard, Stockard, & Tusler, 2003). Establishment of a pain management monitoring system would require that consistent methods be clearly defined to measure pain management in target patients and in the success/failure of treatment.

There are several ways to define the target patients who need pain management. The ideal way, which would be to include all patients with cancer who suffer from any kind of pain, would require a process of asking all patients with cancer (perhaps before definitive diagnoses are made) about their pain, since some patients may not have discussed their pain with their health providers. An alternative way may be to target only patients under some type of pain management or patients taking analgesic drugs. This way overlooks patients with pain not recognized by health providers, and thus fails to consider providers’ ability or efforts to thoroughly detect patients’ suffering. On the other hand, because this way does not rely on obtaining patients’ reports, it provides a more defined range of target patients and saves the time and effort of interviewing individual patients about pain.

While the theoretical limitation associated with focusing on patients already being treated for pain is clear, an important unanswered question is: How much labor can we expect to save by limiting the number of target patients? We have found no studies in the literature that report the percentage of patients with cancer being treated for pain in Japan. Although surveys from other countries have reported their prevalence of pain (Breivik et al., 2009; van den Beuken-van Everdingen et al., 2007) and proportion of treatment for moderate to severe pain (Breivik et al., 2009), they have not focused on the frequency of prescribing pain medications associated with resource allocation for monitoring of pain management in hospitals. The purpose of this study was to gain insight into the current status and recent time trend of the use of pain medications in Japan. We analyzed a large database of insurance claims from multiple employer-sponsored insurance companies.

## 2. Methods

### 2.1 Dataset

#### 2.1.1 The Health Insurance System in Japan

We analyzed insurance claims data sets from 8 employer-sponsored insurance companies. In Japan, all residents have health insurance from either their employment or their place of residence. Many large companies work with associated insurance companies (1435 insurance companies as of April 2012 (National Federation of Health Insurance Societies (Kenporen), 2012)). Relatively small companies who do not work with associated insurance company provide coverage through the Japan Health Insurance Association. Unemployed or retired persons and persons aged 75 years or older have coverage based on their place of residence from city or region-based insurance entities, respectively.

Health services are reimbursed on a fee-for-service basis according to a nationally defined fee schedule. The healthcare facilities submit claims every month for each patient. The claims list all the services and medications provided to patients in the facility as well as the diagnoses corresponding to those services and medications. For patients who receive drug prescriptions, claims for the medications are submitted by the pharmacy that has dispensed the prescription. These pharmacy claims also contain the names of the prescribing facilities, thus providing links to the prescribing claims.

#### 2.1.2 Study Sample

For our study, eight insurance companies provided data from a total of 750 000 members consisting of the employees of affiliated companies and their dependents. Among them, three insurance companies provided claims from January 2005 to December 2009 and five provided claims from January 2008 to December 2009. The claims from these eight insurance companies included a total of 84652 patients with any type of cancer diagnosis, including tentative diagnoses. To avoid ambiguity of diagnosis on the insurance claims, we analyzed data on patients who had received anti-cancer treatment for the five major cancers in Japan, namely, breast, colorectal, stomach, lung, and liver cancers. Anti-cancer treatment included surgery, chemotherapy, hormone therapy, and radiation therapy. We excluded patients who had undergone only endoscopic treatment, because we suspected that cancer painmay not have been an issue for them.

#### 2.2 Statistical Analyses

Analgesic drugs were classified according to the World Health Organization Pain Control Ladder (World Health Organization., 1996); non-steroidal anti-inflammatory drugs (NSAIDs) including acetaminophen, weak opioids (ie, codeine, dihydrocodeine, tramadol, and pentazocine), and strong opioids (ie, morphine, oxycodone, fentanyl, pethidine, and buprenorphine). Low-dose aspirin (100mg/tablet) and the codeine contained in cold medicines were not regarded as painkillers. For each month during the study period, the proportion of patients with cancer who received each type of drugs was recorded.

The proportion of analgesic prescriptions were compared between patients’ treatment phases (ie, after surgery, after chemotherapy, and after radiation) and primary cancer site. Definition of the treatment phase was based on the last anti-cancer therapy. For example, patients who received surgery followed by chemotherapy (at a later time) were considered to be “after surgery” for the period between surgery and chemotherapy, and “after chemotherapy” after the chemotherapy had been received. Primary cancer sites were determined on the basis of both the cancer treatment and diagnosis recorded on insurance claims. For those patients who had undergone surgery, including site-specific intervention (eg, radio frequency ablation therapy to the liver), the primary site of cancer was considered to be the target organ. For those patients who had received only systemic chemotherapy, where the target cancer had not been clearly established, or radiation therapy where the insurance reimbursement code was the same across different target sites, the diagnoses in the insurance claims were accepted as they had been recorded. The differences in the proportions were statistically tested using the chi-square tests.

The trend of prescribing analgesic drugs for cancer patients was described as the proportion of patient-prescribed analgesic drugs among the cancer patients who had used any health services during a given month. The person-month was the unit of analysis. The change in the trends was analyzed graphically. Also the beta coefficients to represent the trend was calculated using linear regression analyses where the percentage of analgesic prescriptions and the time variable were the dependent and independent variables, respectively, assuming the linearity of the relationship. Because the assumption of homoscedastic errors did not hold for some regression models, the robust standard errors were calculated with the White correction. No correlation between error terms and the independent variable was confirmed. All analyses were performed using Stata 11.2 (StataCorp LP, College Station, Texas).

## 3. Results

A total of 6656 patients had one of the five major cancers on the health insurance claims, among whom 2585 patients received treatment with surgery, chemotherapy, and/or radiotherapy during the study period, and thus were entered into the analyses. Patient characteristics are presented in Table 1. Average patient age was 53.4 years (Standard deviation: 10.6); 57.7% of patients were female. The most common cancer was breast cancer (n =923 [35.7%]), followed by colorectal cancer (n =615 [23.8%]) and stomach cancer (n =465 [18.0%]). The average duration of the observation period (ie, from first cancer treatment to last visit) was 33.8 months.

**Table 1:** Patient characteristics

Age		
<20	21	( 0.8% )
20-39	243	( 9.4% )
40-59	1619	( 62.6% )
60-69	548	( 21.2% )
>70	154	( 6.0% )

**Gender**		
Female	1491	( 57.7% )

**Cancer site**		
Breast	923	( 35.7% )
Colorectal	615	( 23.8% )
Liver	179	( 6.6% )
Lung	412	( 15.9% )
Stomach	465	( 18.0% )

**Treatment received**		
Surgical Intervention	1586	( 61.4% )
Chemotherapy	1629	( 63.0% )
Radiation	594	( 23.0% )

Tables 2 and 3 show the percentages of patients receiving analgesic prescriptions every month by treatment phase and by site of cancer, respectively. Overall, 22.9% of patients who used healthcare each month received analgesic prescriptions (Table 2). Analyses for each drug class revealed that NSAIDs or acetaminophen and opioids were prescribed in 19.8% and 9.1% of the patients, respectively. Strong and weak opioids were prescribed 6.2% and 4.0% of visits, respectively. When we separated patients by treatment received, patients after chemotherapy were most frequently prescribed analgesics (23.7%), while opioids were most frequently prescribed for patients after radiation therapy (9.8%). The analysis by site of cancer revealed that patients with lung cancer were more likely to receive analgesics (overall, 33.3%) than patients with other types of cancer (Table 3).

**Table 2:** Average proportion of analgesic prescriptions every month by treatment phase

	Overall	After surgery	After chemotherapy	After radiation	P value
Any analgesics	22.9%	21.4%	23.7%	22.8%	<0.001
ACA	1.9%	1.9%	2.2%	2.6%	<0.001
ACA/NSAIDs	19.8%	18.8%	20.7%	20.8%	<0.001
Opioid	9.1%	6.7%	9.4%	9.8%	<0.001
Weak opioid	4.0%	4.2%	3.8%	4.3%	0.16
Strong opioid	6.2%	3.6%	6.7%	6.5%	<0.001

Abbreviations: ACA acetaminophen; NSAID non-steroidal anti-inflammatory drug.

**Table 3:** Average proportion of analgesic prescriptions every month by site of cancer

	Breast	Colorectal	Liver	Lung	Stomach	P value
Any analgesics	20.0%	20.8%	23.8%	33.3%	17.1%	<0.001
ACA	1.9%	1.9%	2.5%	2.5%	1.9%	0.01
ACA/NSAIDs	18.4%	17.7%	19.9%	28.7%	14.4%	<0.001
Opioid	4.2%	8.9%	9.6%	17.6%	7.0%	<0.001
Weak opioid	2.1%	3.5%	7.0%	6.1%	2.8%	<0.001
Strong opioid	2.4%	6.6%	3.1%	13.2%	5.3%	<0.001

Abbreviations: ACA acetaminophen; NSAID non-steroidal anti-inflammatory drug.

**Figure 1:**
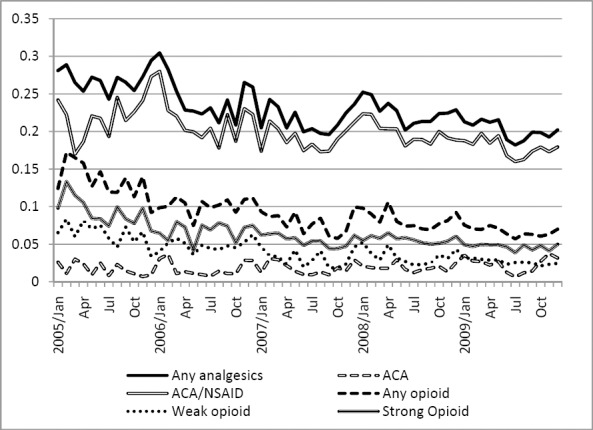
Trend of analgesic prescriptions over observation period

Figure 1 shows a decrease in the percentage of patients who received analgesic prescriptions over the observation period (ie, January 2005 to November 2009). Table 4 shows that analgesic prescriptions decreased by 0.13% per month as calculated via regression analysis.

**Table 4:** Monthly decrease of proportion of analgesic prescriptions(linear regression analyses)

	Beta	(95% CI)		P value
Any analgesics	-0.13%	-0.15%	-0.11%	<0.001
ACA	0.01%	0.00%	0.02%	0.13
ACA/NSAIDs	-0.08%	-0.11%	-0.05%	<0.001
Opioid	-0.13%	-0.15%	-0.10%	<0.001
Weak opioid	-0.07%	-0.09%	-0.06%	<0.001
Strong opioid	-0.09%	-0.11%	-0.07%	<0.001

Abbreviations: ACA acetaminophen; NSAID non-steroidal anti-inflammatory drug.

## 4. Discussion

Our study, using health insurance claims data from employee-sponsored insurance companies, showed that about one fourth of the patients treated for the five major cancers in Japan received analgesic prescriptions. Since the patients counted in our study were in treatment, the true prevalence of pain that include patients with pain but not in treatment among these patients will be higher. Restricting our target group of patients to patients taking analgesic medications will facilitate selection of patients for evaluating the performance measurement of pain management. By selecting patients in treatment for this evaluation, we will be getting by with only one-fourth to one-fifth of the all the patients. In addition, restricting our target group to patients taking analgesic medication is likely to be more efficient for systematic evaluation purposes than identifying and assessing patients with pain from all patients with cancer.

It must be noted that our study neither implies that identifying patients with untreated pain is of little value nor does it advocate limiting target patients for the monitoring of pain management. Although the prevalence of pain among Japanese patients with cancer is unknown, the prevalence of analgesic prescriptions is much lower than prevalence of pain itself reported in other countries. One systematic review showed that about half of all diseases stages and a third of patients after curative treatment reported pain (van den Beuken-van Everdingen et al., 2007). A population-based survey from Europe and Israel showed that 74% of patients with cancer reported pain (Breivik et al., 2009). Although the patients with cancer in our study were younger and in better condition than the average patient with cancer (Center for Cancer Control and Information Services, National Cancer Center, 2011), the gap between the prevalence of pain among patients with cancer and our finding that 22.9% of patients received analgesics may be an indication of the undertreatment of pain in Japan. This gap underscores the importance of detecting pain among cancer patients.

We need to implement better pain management for cancer pain nationwide. While we are not satisfied with a limited target patient group for assessment in planning a nationwide system, we understand that starting with a limited group is a realistic option. Uniform application of the assessment with clear definition is essential to encourage improvement. Even if we start small, we will eventually assess all patients with cancer who are experiencing pain and ensure that they have access to pain management and appropriate treatment.

In order to work toward a nationwide pain management system for cancer pain, we need to be cautiously aware of the nature of the data to be used and the findings on opioid consumption in Japan. The decreasing trend of patients receiving analgesic drugs in the observed data may be associated with the composition of patients in treatment phases shifted from acute-phase dominant to chronic follow-up phase dominant over time. Because we enrolled patients from the month in which they began cancer therapy and followed up later, patients under observation in the early years of the study period were usually enrolled right after the treatment, making them more likely to receive analgesic medications for pain that arose from the anti-cancer treatment (eg, wound pain after surgery, dermatitis after radiation therapy). In the later years of the study we observed patients both in regular follow-up and patients receiving acute treatment. Thus, a larger proportion of more stable patients in regular follow-up may have caused the overall proportion of analgesic prescriptions to decrease. Nonetheless, the decrease was not steep and therefore did not greatly influence our findings.

The impact of the insurance claims on our findings also warrants mention. First, the insurance companies that provided the data were employer-sponsored. As such, they exclusively enroll employees and employees’ dependents. We suspect therefore that our target patient group tended to be younger than the average cancer patient. In fact, while the national statistics on the hospital-based cancer registries showed that most cancer patients to be in their 60s and 70s (Center for Cancer Control and Information Services & National Cancer Center, 2011), most of the patients with cancer in our studies were their 50s and 40s. Second, the accuracy of the diagnosis may be questionable. Since insurance claims place more emphasis on consistency between diagnoses and services provided than clinical accuracy, determining whether a diagnosis is tentative or final is difficult. Third, claims data do not describe the symptoms for which the drugs were prescribed. Therefore, we cannot determine whether NSAIDs were prescribed for pain, fever, or some other anti-inflammatory malady. Fourth, claims submitted to insurance companies lack information on services out of the fee-for-service reimbursement. In 2003 the Japanese health insurance system started paying per-diem based on predefined information from diagnosis-procedure groups in 82 participating hospitals. The number of participating hospitals gradually increased, and in 2011, a total of 1447 hospitals (19% of total) in Japan were participating (Bureau of Health Insurance, Ministry of Health, Labor, & Welfare, 2012). Most services and medications provided during hospitalization to these hospitals were not captured in regular insurance claims, increasing the likelihood of underestimating analgesic use during hospitalization. Fifth, we limited our analyses to the patients who received therapy for the five major cancers in Japan. By limiting the cancer type to the five major cancers, we could match the match the claim diagnoses with the treatment. This enabled us to exclude patients with a tentative diagnosis who turned out not to have cancer later or inactive diagnosis that was treated could remain on the claims even after treatments were over. However, in real clinical practice, the target for pain management should include all cancer types. We need to bear in mind that the results may have been different if we included all cancer types. Finally, since our data are derived from health insurance companies, the number of patients per hospital was small for many hospitals. Given that taking analgesic prescriptions in small denominators is not likely to produce stable results, we did not perform analyses at the level of individual providers.

## 5. Conclusion

Our study showed the prevalence of analgesic prescriptions among five major cancers in Japan. When planning for a system that monitors the performance of pain management, it is important to balance the resources used with the range of the target for the measurement. The frequency of analgesic prescription provided information for an evidence-based discussion on how to restrict or broaden the target population for monitoring using available resources. Even if we decide to begin systematic evaluation with a smaller target patient group (ie, patients already taking analgesics), we will do so keeping in mind that our ultimate goal is to provide pain relief to all patients with cancer in our country.
